# Foot-and-Mouth Disease: Optimization, Reproducibility, and Scalability of High-Yield Production of Virus-Like Particles for a Next-Generation Vaccine

**DOI:** 10.3389/fvets.2020.00601

**Published:** 2020-09-23

**Authors:** Ana Clara Mignaqui, Alejandra Ferella, Brian Cass, Larissa Mukankurayija, Denis L'Abbé, Louis Bisson, Cintia Sánchez, Romina Scian, Sabrina Beatriz Cardillo, Yves Durocher, Andrés Wigdorovitz

**Affiliations:** ^1^Instituto de Investigaciones Forestales y Agropecuarias de Bariloche, IFAB, INTA-CONICET, San Carlos de Bariloche, Argentina; ^2^Instituto de Virología e Innovaciones Tecnológicas, IVIT, INTA-CONICET, INCUINTA, Buenos Aires, Argentina; ^3^Human Health Therapeutics Research Center, National Research Council Canada, Montreal, QC, Canada; ^4^Biogénesis-Bagó S.A, Buenos Aires, Argentina

**Keywords:** FMDV, VLPs, mammalian cells, transient gene expression, emergency vaccine

## Abstract

Inactivated Foot-and-Mouth Disease (FMD) vaccine has proven to be effective in the control of the disease. However, its production has some disadvantages, including the costly biosafety facilities required for the production of huge amounts of growing live virus, the need of an exhaustive purification process to eliminate non-structural proteins of the virus in the final formulations in order to differentiate infected from vaccinated animals and variable local regulatory restrictions to produce and commercialize the vaccine. Thus, a novel vaccine against FMD that overcome these restrictions is desirable. Although many developments have been made in this regard, most of them failed in terms of efficacy or when considering their transferability to the industry. We have previously reported the use of transient gene expression in mammalian cells to produce FMD virus-like particles (VLPs) as a novel vaccine for FMD and demonstrated the immunogenicity of the recombinant structures in animal models. Here, we report the optimization of the production system by assaying different DNA:polyethylenimine concentrations, cell densities, and direct and indirect protocols of transfection. Also, we evaluated the reproducibility and scalability of the technology to produce high yields of recombinant VLPs in a cost-effective and scalable system compatible with industrial tech-transfer of an effective and safe vaccine.

## Introduction

Foot-and-Mouth Disease (FMD) is a highly contagious and threatened disease of cloven-hoofed animals, endemic in many parts of the developing world (1). It is one of the most important animal health concerns from an economic point of view and continues to pose a serious threat to farmers, livestock industries and governments. The presence of the disease in developing countries results in severe restrictions to the international trade and an outbreak in FMD-free countries can cause billionaire losses. Vaccination programs in endemic countries or those FMD free with vaccination consider annual or semi-annual vaccination with conventional, inactivated vaccines. The policy of applying vaccination to respond to incursions in FMD free countries, known as “vaccination to live,” gained acceptance as a result of public questioning after the slaughter of millions of animals due to large outbreaks in Europe in 2001 (2, 3). Vaccination is useful in many possible scenarios: for prevention or control of an outbreak in FMD-free areas and for the control of the disease in endemic regions.

The currently marketed inactivated virus vaccine consists of chemically inactivated virus formulated, in most of the cases, in oil-based adjuvant (4). For this type of vaccines, baby hamster kidney-21 (BHK-21) cells are grown in large scale bioreactors and then are infected with a specific Foot and Mouth Disease Virus (FMDV) strain. The virus obtained after this process is inactivated with binary ethyleneimine (BEI) and after purification can be used for vaccine formulation. Although the inactivated vaccines are effective to control the disease, they have many disadvantages that have prompted the development of novel vaccines (5, 6). These disadvantages include the need of costly biosafety facilities that require constant investment in manufacturing plant up-grades and qualified personnel as well as strict controls to eliminate the possibility of viral incomplete inactivation. In addition, rigorous purification steps during production are needed to avoid the presence of FMD viral non-structural proteins in the final formulation and thus later be able to differentiate between infected and vaccinated animals. Another important issue is that related to the policies of regulatory sanitary agencies across many FMD-free regions and countries, which restrict the production of growing live virus in their mainland. Finally, other key obstacles include the high variability of the virus and the lack of cross protection between serotypes or in some cases between members of the same serotype.

FMDV is a non-enveloped positive-sense single-stranded RNA virus, member of the Picornaviridae family and genus Aphthovirus (7). FMDV is classified into seven serotypes: A, O, C, SAT1, SAT2, SAT3, and ASIA1, and into many subtypes within each serotype (8). FMDV structural proteins are encoded by the polyprotein P12A and assembled to form the icosahedral capsid after protease 3C cleavage of P12A into mature VP0, VP3 and VP1. Finally, after RNA encapsidation, VP0 is cleaved into VP2 and VP4 and the infectious viral particle is assembled. One copy of each structural protein forms a protomer (5S), five protomers (12S) form a pentamer and twelve pentamers form the empty capsid (75S). Most of the novel developments in FMD vaccines are based on recombinant empty capsids (also referred as virus-like particles, VLPs). These VLPs are promising alternative antigens for vaccine development because they mimic the viral structure and have the complete repertoire of epitopes in a particulate and repetitive form but lacking the infectious RNA (9). Many expression systems and recombinant strategies, including baculovirus expression in cells and larvae, bacterial expression using SUMO technology, DNA vaccines and viral vector vaccines -especially using adenovirus-, have been developed to produce a novel FMD vaccine based on VLPs (10). Moreover, several researchers have already demonstrated the immunogenicity of this recombinant structures produced in various expression systems and with different strategies (11–18). Among these strategies, the most advanced technologies include the use of a baculovirus expression system in insect cells and adenovirus vector vaccines. Using the baculovirus expression system, a novel mutation in the VP2 sequence of FMDV serotype A has been reported to produce thermostable VLPs which is especially helpful in some developing countries where maintaining the cold chain for vaccine distribution can be complicated (14). Regarding viral vector vaccines using adenovirus technology for serotype A, there is already a license in the USA for emergency use in case of an outbreak (5, 18, 19). We have previously demonstrated that VLPs based on A2001 Argentina strain, produced using transient gene expression (TGE) in suspension-growing cells, were able to elicit an immune response in a mouse model with a 100% protection after viral challenge, a response comparable to the one obtained using a similar amount of inactivated virus (20). We have also recently demonstrated the immunogenicity of FMDV VLPs in cattle (21).

The traditional way of producing recombinant proteins in mammalian cells is the development of stable cell lines (22, 23). However, toxic proteins like protease 3C do not allow the development of stable cell lines. In this context, TGE is a simple and fast technology for the production of recombinant proteins, which was developed to produce mg of proteins for the first steps in clinical trials and thus represents the strategy of choice for mammalian cell expression of toxic proteins (24). In the case of FMD novel vaccines, TGE has some advantages because the process itself is quite similar to the current production of the inactivated virus but the preparation of viral seeds and infection of cells are replaced by plasmid production and transfection, respectively. The possibility of fast cloning the P12A sequence of different serotypes into the expression plasmid makes TGE a promising technology for the development of a novel vaccine against FMD, especially in emergency scenarios where fast responses are required.

Although many of the efforts made to develop a novel VLP-based vaccine for FMD have shown promising results, the challenge is still to have a novel vaccine that is as effective as the currently marketed inactivated one but produced with simple and scalable technology to encourage the tech-transference steps required to move from bench to market. These key aspects of novel strategies are scarcely reported. Considering that it is now generally accepted that VLPs are the best recombinant antigens to produce a novel vaccine against FMD, it is desirable to show the simplicity and scalability of the different recombinant technologies in order to make them attractive to the industry. Thus, the aim of this work was to make an effort in this regard by moving forward to increasing the yield of VLPs from TGE by using codon-optimized plasmids and assaying different DNA:polyethylenimine (PEI) concentrations and cell densities. We also assayed direct and indirect protocols, evaluated the addition of an antiapoptotic gene in the transfection mixture, and focused on the reproducibility and scalability of the already reported technology. Finally, we performed vaccine formulation assays to study the antigen integrity and stability throughout this process.

## Materials and Methods

### Cells and Viruses

The human embryonic kidney 293 cell line stably expressing a truncated Epstein–Barr virus Nuclear Antigen-1 (293-6E cells) was grown in suspension in serum-free F17 medium (Gibco), as previously described (20). Cells were grown in a humidified incubator at 37°C with 5% CO_2_, with agitation at 120 rpm.

### Plasmids

FMDV DNA sequences were cloned under the CVM promoter in the pTT5 vector (25). The pTT5-P12A3C plasmid encoding wild type sequences from FMDV A2001 Argentina strain was previously constructed (20). The P12A sequence from FMDV A2001 was codon-optimized for mammalian expression by GenScript (Supplementary Data) and the synthetic gene encoding P12A was subcloned in the pTT5 vector with protease 3C in tandem by digesting the pTT5-P12A3C wild type with the restriction enzymes NheI and BstBI. A Kozak sequence was included immediately upstream the start codon. pTT22-hAktDD encoding a constitutively active Akt mutant was co-transfected as described previously (23, 26). *Escherichia coli* (DH5α) grown in Circle Grown medium (MP Biomedicals, Solon, OH, USA), supplemented with 50 μg/mL ampicillin was used for plasmid production. The plasmid was purified using MAXI prep columns (Qiagen, Mississauga, ON, Canada) according to the manufacturer's instructions. The A260/A280 ratios were measured and only plasmid preparations with ratios between 1.75 and 2.00 were used.

### Production of Recombinant VLPs

293-6E cells were transfected using polyethylenimine (LPEI-MAX) (Polysciences, Warrington, PA, USA), as previously reported (20). Briefly, cells were seeded 2 days before transfection at 0.45–0.5 × 10^6^ cells/mL. The day of transfection, viability and cell densities were determined. Only cells with viability >95% and densities between 1.5 and 2 × 10^6^ cells/mL were transfected. For the indirect protocol: the plasmid and PEI were diluted in complete medium and PEI was added to the DNA dilution and mixed. After incubation at room temperature for 3 min, the DNA:PEI mixture was added to the culture. For the direct protocol: the plasmid and PEI were diluted in complete medium and added to the cell cultures (27). Shake flasks of different volumes (125, 250, 500 mL, and 1 L) and a 10 L Bioreactor were used for the transfection. Cells were harvested, centrifuged and resuspended in lysis buffer (50 mM HEPES pH 7.4, 150 mM NaCl, 1% Thesit, 0.5% NaDeoxycholate). The use of PEI for transfection may be covered by existing intellectual property rights, including the US Patent 6,013,240, the European Patent 0,770,140, and foreign equivalents, for which further information may be obtained by contacting licensing@polyplus-transfection.com.

### Cell Counts

Cell density and cell viability were measured using an automated cell counter, Cedex Analyzer, based on the trypan blue exclusion method (Roche, Laval, Qc).

### Recombinant Protein Analyses

For Western Blotting analysis, lysates were separated by sodium dodecyl sulfate polyacrylamide gel electrophoresis (SDS-PAGE). Then, proteins were transferred onto a polyvinylidene difluoride membrane, blocked and then incubated with anti-FMDV guinea pig serum (1/500) produced in house using wild type inactivated FMDV A2001 Argentina strain. After several washes, membranes were incubated with horseradish peroxidase-conjugated anti-guinea pig goat serum (1/1,000) (KPL). The reaction was visualized with an enhanced chemiluminescence method in a GBox (Syngene). The VLPs were quantified by enzyme-linked immunosorbent assay (ELISA). For coating of microtiter plates (Maxisorp), a polyclonal anti-FMDV serum made in rabbit (1/3,000) was diluted in carbonate-bicarbonate buffer, pH 9.6, and incubated at 4°C overnight. The washing steps were done with phosphate-buffered saline (PBS) 0.1% Tween-20 and blocking for 30 min at 37°C with 5% normal equine serum in PBS 0.1% Tween-20. Samples were incubated at 37°C for 1 h. For the generation of standard curves, known amounts of inactivated FMDV were serially diluted and added to the wells. Plates were then incubated for 1 h with a polyclonal anti-FMDV serum made in guinea pig (1/3,000), followed by horseradish peroxidase-conjugated anti-guinea pig goat serum (KPL). Then, tetramethylbenzidine was added and, 5 min later, the reaction was stopped with sulfuric acid 12%. Absorbance at 450 nm (A450) was recorded in a microplate reader (Thermo Scientifics MultiskanFC). For gradient assembly, 1 mL of 45, 35, 25, and 15% sucrose solutions (W/V) was added to ultracentrifuge tubes Ultra-Clear tubes 1/2 × 2 in (13 × 51 mm). The most concentrated solution was located at the bottom of the tube and the most diluted at the top. Samples were added on top of the gradient. The tubes were centrifuged in a Beckman Optima-LP X-100 Ultracentrifuge using a SW 55 Ti rotor for 2 h at 45,000 rpm at 4°C, acceleration: 9, deceleration: 9. Once the centrifugation was completed, 0.5 mL aliquots were collected. Aliquots were tested for ELISA-specific FMDV protein. Both the gradient assembly and the sample collection were performed manually.

### Vaccine Formulation

Cells were harvested, centrifuged at 4,000 g and the supernatant was discarded. Pellets were resuspended in Tris-salt buffer and subjected to three freeze-thaw cycles at −80/25°C. Finally, the lysate was clarified by centrifugation and VLPs were quantified by ELISA as described above.

For the vaccine formulation, a water-in-oil (W/O) single emulsion was prepared by adding the formulated aqueous phase containing the VLPs to the oily phase containing mineral oil and emulsifier agents. The mixture was then emulsified using an UltraTurrax homogenizer for 8 s at full speed. The particle size distribution of the final emulsion was analyzed by laser diffraction using the Mastersize 2000 (Malvern). To obtain the aqueous phase for analysis, the emulsion was disrupted by adding 1 volume of chloroform in a 15 mL tube. The blend was mixed gently up and down for 1 min and centrifuged for 10 min at 4,000 rpm. The aqueous phase was collected and the procedure of adding chloroform was repeated. The VLPs in the recovered aqueous phase were evaluated by sucrose gradient and quantified by ELISA as described above.

## Results

### Optimization of FMDV VLPs

First, we compared the VLP yield obtained using wild type viral sequences and codon-optimized synthetic sequences for mammalian cells encoding for FMDV proteins. The expression levels achieved with the optimized sequence were slightly higher than those achieved with the wild type sequence, although the codon adaptation index changed from 0.74 to 0.95 (data not shown). So, after that, all the experiments shown were performed with the codon-optimized sequence.

**Figure 1:**
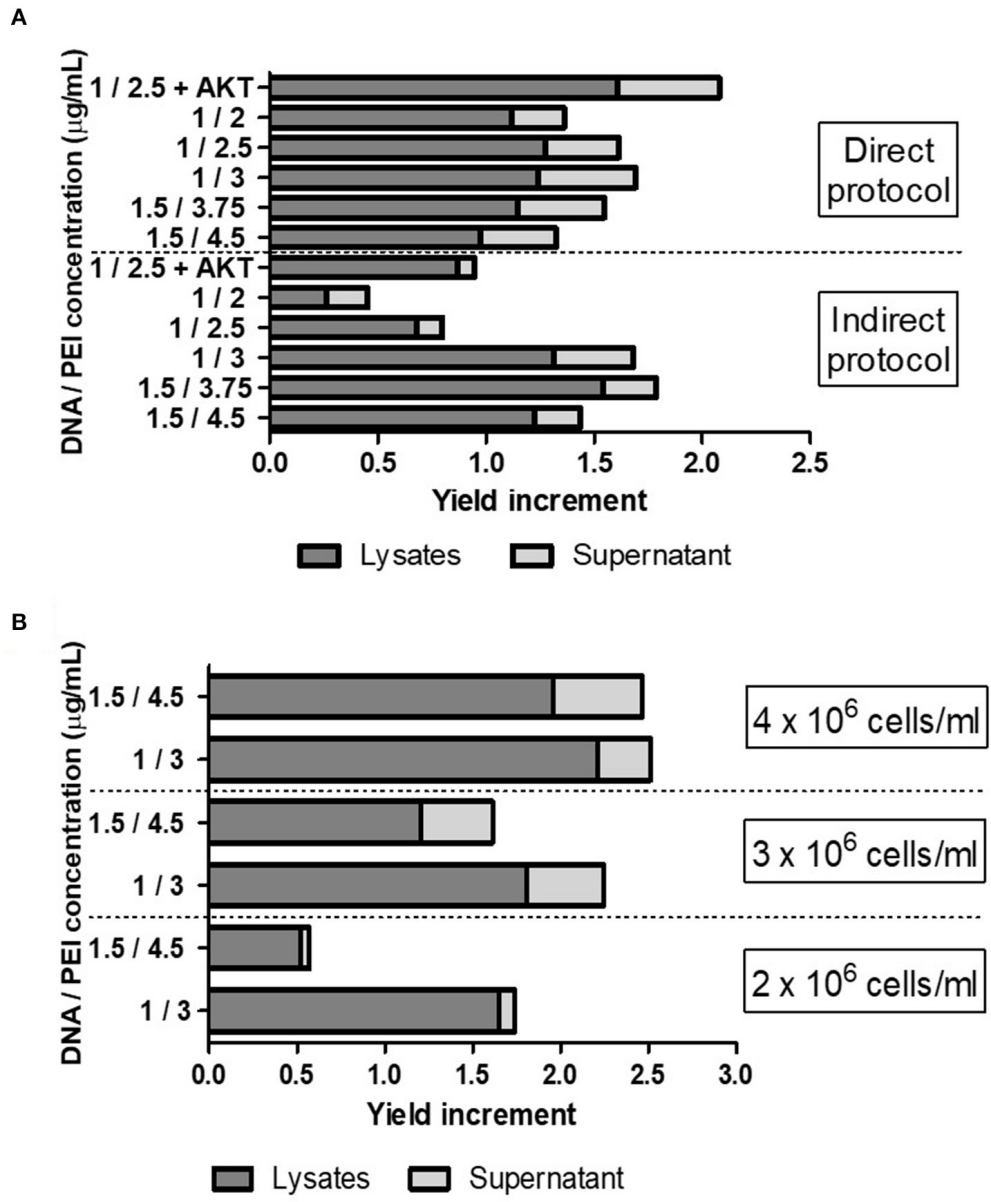
**(A)** Evaluation of different DNA:PEI concentrations (μg/ml) in both direct and indirect protocols and effect of the addition of an antiapoptotic gene AKT in the transfection mixture. **(B)** Evaluation of different cell densities using the pTT5-P12A3C plasmid plus AKT, using different DNA:PEI concentrations. Cell cultures with 3 and 4 × 10^6^ cells/mL were produced using cultures with a cell density of 2 × 10^6^ cells/mL and centrifugation steps before transfection. The increase in VLP yield was measured by Western Blotting analysis of the VP0 band intensity, comparing each transfection with the transfection condition previously published in Mignaqui et al. (20).

To further optimize the VLP yield achieved by TGE, we evaluated different DNA:PEI concentrations in both direct and indirect protocols, using the pTT5-P12A3C plasmid. Also, considering the toxic effects of protease 3C on cells, we tested the effect of the addition of an antiapoptotic gene (AKT) in the transfection mixture (Figure 1A) (23, 28). Among all the conditions evaluated, the highest increase in yield was around 2-fold higher than that obtained by the use of the pTT5-P12A3C plasmid in previously reported conditions and was achieved when the antiapoptotic gene was added in the transfection mixture. Overall, indirect transfection protocols yielded higher recombinant VLPs levels. However, using a direct protocol with a DNA:PEI mixture of 1.5:3.75 μg/ml we obtained 50% of increase in the VLP yield per mL of cell culture. The advantage of the direct protocol is that the DNA:PEI mixture step is avoided because the procedure implies the direct addition of DNA and PEI to the cell culture.

Then, we evaluated different cell densities and different DNA:PEI ratios and used a combination of pTT5-P12A3C plus 15% of a plasmid encoding AKT for the transfection, which allowed us to further increase the recombinant VLP yield (Figure 1B). Indeed, the highest yield was achieved when the highest number of cells was used.

### Reproducibility and Scalability of FMDV VLPs

**Figure 2:**
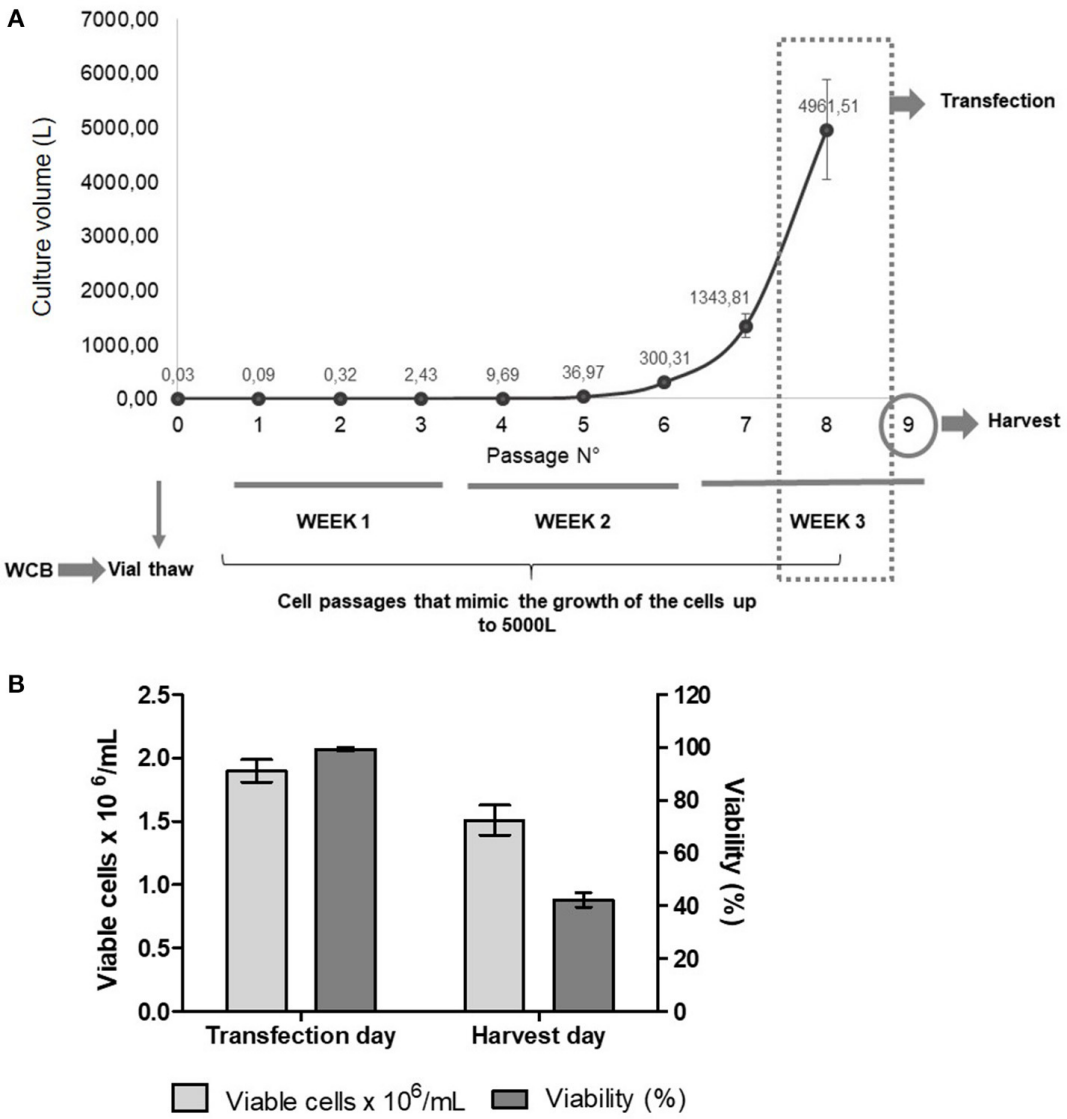
**(A)** Experimental design of the reproducibility assay. After vials were thawed, cells were grown for 3 weeks, mimicking the passages needed to achieve a volume of around 5,000 L. After 7 passages, cells were transfected and, at 48 hpt, cells were harvested for protein analysis. WCB, Working cell bank. **(B)** Average of viable cells × 10^6^/mL and viability (%) of cell cultures on the day of transfection and on the harvest day (48 hpt).

**Figure 3:**
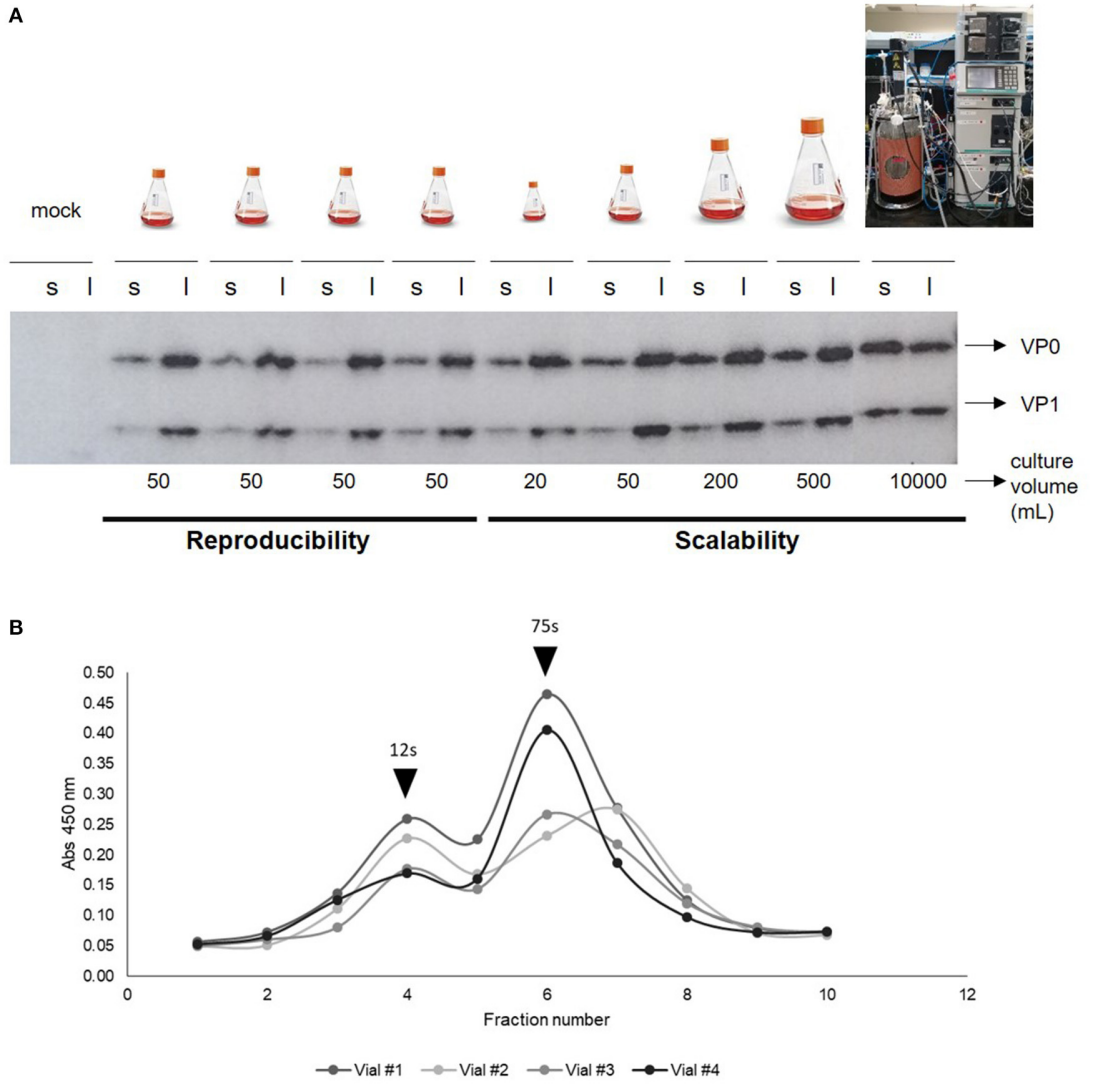
**(A)** Western Blotting of cell lysates (l) and supernatants (s) after 48 h of independent transfections of different culture volumes (10 L, 500, 200, 20, and 50 mL) with FMDV-encoding plasmids. Primary antibody: anti-FMDV polyclonal serum made in guinea pig (1/500) and secondary antibody: anti-guinea pig peroxidase (1/10,000). **(B)** Cell lysates of cultures of independent transfections of 50 mL at 48 hpt analyzed by sucrose gradient to evaluate VLPs formation. Black arrows indicate 12s or 75s peak, corresponding to structural proteins assembled in pentamers or in complete empty capsids, respectively.

To study the reproducibility and scalability of our technology, we prepared a working cell bank with vials with 10 million cells per mL. Then, independent assays were performed by starting cell cultures using four cryovials of the working cell bank. After thawing the cryovials, cells were grown for three weeks at low scale (final volume 50 mL), mimicking the cell passages needed to achieve a 5,000 L culture (Figure 2A). Only cells from one vial were also grown to different volumes: 20, 50, 200, 500 mL, and 10 L. Moreover, we performed the transfection by using independent DNA:PEI mixtures. Cell counts and viability were recorded during cell growth, on the day of transfection, and on the harvest day (Figure 2B). VLP expression was analyzed by ELISA, Western Blotting and sucrose gradient (Figures 3A,B). The growth curves and viability of the different cultures during cell passages during the 3-week period were similar to each other (data not shown). On the transfection day, cell density was between 1.5 and 2 × 10^6^ cells/mL and viability >95%, respectively, in all cell cultures. When cells were harvested at 48 h post-transfection (hpt), cell density and viability were similar in all the flasks, with a viability of 42% in average, which is very low and confirms the toxic effects of FMDV proteins, especially protease 3C, on cells. When 20 or 50 mL were transfected, the distribution of VLPs between the supernatant and the lysate was around 20 and 80%, respectively. However, when the transfection volume increased, the specific protein detected in the supernatant also increased, being around 60% in the 10 L bioreactor. Probably, the shear stress in the 10 L bioreactor was stronger than in the 50 mL transfection, thus, if the bioreactor is used, the harvest time should be further optimized. Interestingly, VLP formation was reproducible in the four 50 mL transfections, as determined by sucrose gradient analysis, with more than 92 ± 8% of the structural proteins assembled in VLPs. Overall, we were able to demonstrate high reproducibility and scalability of the technology for the production of FMDV VLPs. The maximum recombinant protein yield in cell lysates was measured by ELISA being 7 ± 0.6 mg per liter of cell culture.

### Vaccine Formulation and Antigen Stability

**Figure 4:**
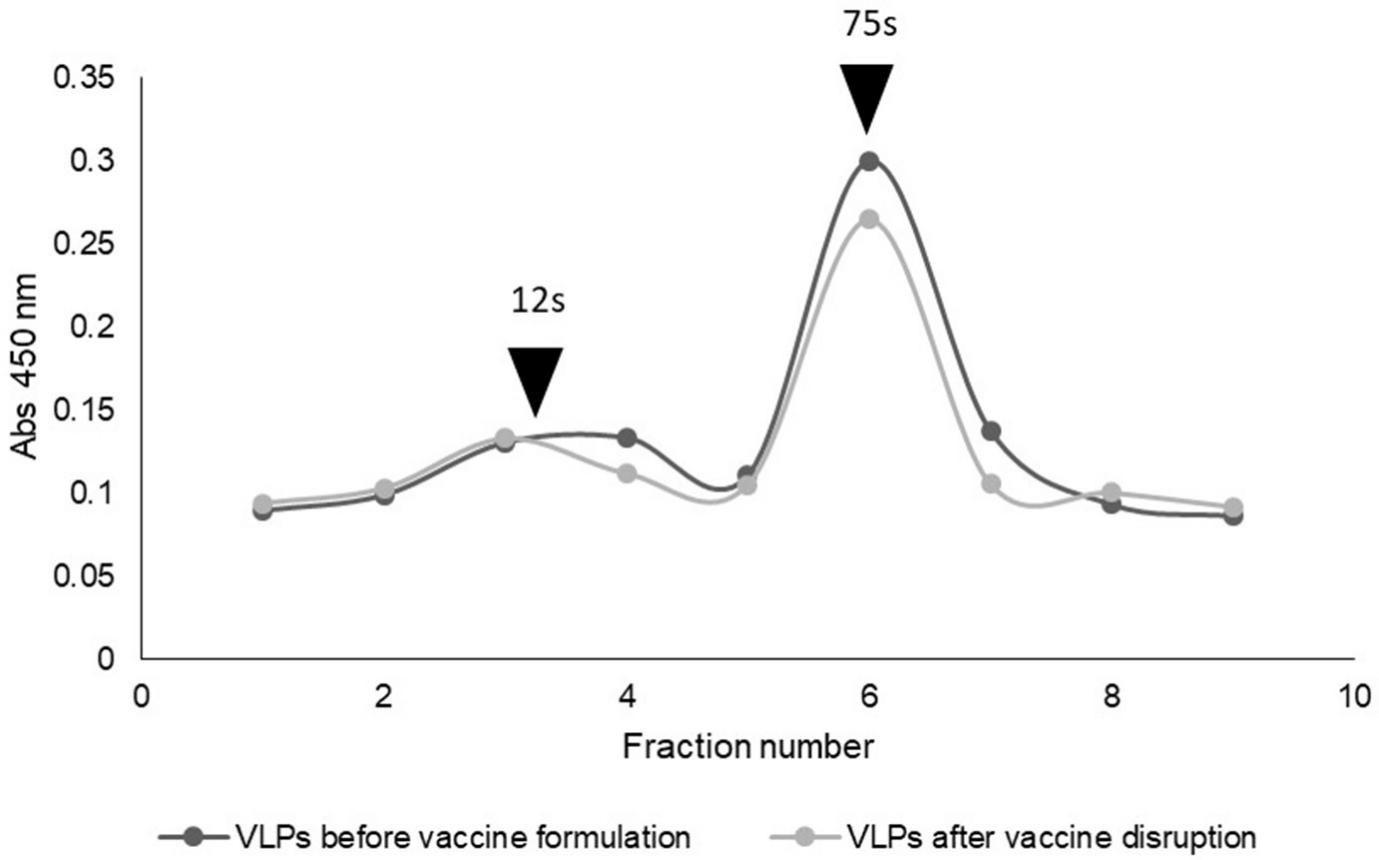
Sucrose gradients showing antigen stability of VLPs within a water-in-oil emulsion vaccine after disruption.

To evaluate the stability of the antigen after the process of vaccine formulation, we prepared a W/O emulsion containing 25 μg of VLPs per dose of vaccine. Then, the emulsion was disrupted by the addition of chloroform, and the VLPs present in the recovered aqueous phase were analyzed by sucrose gradient and quantified by ELISA (Figure 4). The percentages of complete empty capsids (75s) and pentamers (12s) were calculated. Immediately after the vaccine formulation and the subsequent disruption of the emulsion, 84% of the antigen was present as complete empty capsids. Thus, we were able to demonstrate that a high proportion of the VLP antigen is stable after the emulsification procedure of vaccine formulation.

## Discussion

FMD, one of the most devastating diseases of livestock, can cause significant economic losses worldwide, and represents the most important limitation to international trade in live animals and animal products. Although the traditional inactivated vaccine has been proved effective, the development of a novel FMD vaccine that is effective, safer and less expensive than traditional vaccines could be of great value. The feasibility of a tech transfer to occur in the veterinary vaccine technology field after a novel vaccine has proven to be effective relies mostly on the simplicity, scalability and cost-effective properties of the proposed technology. Regarding FMD, many efforts have been made to develop a novel vaccine that overcomes the need of producing huge amounts of growing live virus in costly biosecurity facilities (5, 6, 10). To move from bench to market, the efforts made to develop a novel vaccine against FMD must focus in production aspects related to regulatory concerns, scalability aspects, and cost-effective technology. Here, we focused in the yield of recombinant VLPs, the production procedure, and the reproducibility and scalability of the technology. Although technical details are necessary for an efficient transfer to large-scale production, there are few reports that address them. Most of the efforts have been made to demonstrate that VLPs are the antigens of choice for a novel vaccine for FMD and currently there is relevant evidence that demonstrates that their particulate structure and the complete presence of viral epitopes make them as immunogenic (or almost as immunogenic) as the virus (11–18).

The technology here described could be a great response for emergency vaccines. When an outbreak occurs, the genomic analysis of the viral strain responsible for the outbreak would be enough to start the production of VLPs after a synthetic gene is ordered and cloned into the expression plasmid. After plasmid production by *E. coli* transformation and purification by anion exchange chromatography, cells are transfected, and VLPs are harvested at 48 hpt, The technology allows the optimization of different aspects like the DNA:PEI ratio or the cell density, and the use of direct protocols not only to increase the yield but also to have a more convenient process. Here, we demonstrated that direct transfection protocols can be used, which is a great advantage in case large volumes are transfected because they avoid the step of forming DNA:PEI polyplexes outside the bioreactor (27). Generally, these polyplexes are made in 5–10% of the final volume transfected. So, if the final volume of transfection is 10 L, the polyplex mixture will range between 0.5 and 1 L. Performing the mixture in a flask up to that volume can be possible but doing so with higher volumes would require an extra reactor. In the present study, increasing cell density was effective in increasing the VLP yield. However, the need to concentrate the cell cultures by a centrifugation step is not suitable for large-scale processes. Another key element that should be further studied is the reproducibility of the amount of VLPs produced. In the four independent experiments here performed, we demonstrated that the amount of VLPs obtained was high. There is little information about this key point in other reported technologies.

All novel reported recombinant technologies avoid the need of growing live FMDV with the reduced need of biosecurity level in the production facilities. However, the issues related to the high variability of the virus have not yet been solved. Interestingly, in the case of TGE, the cloning of P12A sequences of different FMDV into the pTT5 vector can be easily achieved to produce different FMDV serotypes. TGE also allows solving problems related to the adaptation of the virus to the cell culture but further research should be done to demonstrate the recombinant expression of more serotypes. Each technology for VLP production has advantages and disadvantages and thus each could be useful in different situations considering the complex scenario of FMD. Here, we demonstrated the robustness of TGE and the many possibilities that can be used to improve the technology to optimize the yield and make it suitable for technology transfer. To go further, we were able to demonstrate that a high proportion of VLPs produced by this technology was stable after being subjected to the emulsification process of water-in-oil vaccines, which is the type of formulations usually used for traditional inactivated FMD vaccines. Although some decrease in the integrity of VLPs was observed after rupture of the emulsion, it must be considered that this procedure can be quite aggressive, and maybe the decrease in VLPs can be an artifact of the techniques used for the analysis. Further studies in target species must be performed in order to continue with the characterization of the vaccine. Overall, these results suggest the potential use of VLPs produced as here described for the development of a next generation vaccine for FMD control.

## Data Availability Statement

All datasets generated for this study are included in the article/Supplementary Material.

## Author Contributions

AM: experimental design, fulfillment, analysis and interpretation of the results, production, characterization, purification and quantification of FMDV empty capsids, and manuscript drafts. BC, LM, DL'A, and LB: transient gene expression experiments and critical revision of the manuscript. AF, CS, RS, and SC: VLPs characterization and critical revision of the manuscript. YD and AW: conception of the work, experimental design, and critical revision of the manuscript. All authors contributed to the article and approved the submitted version.

## Conflict of Interest

SC, RS, and CS, who are employees of Biogénesis Bagó, declare that their judgment and objectivity were not biased by their contractual condition. The remaining authors declare that the research was conducted in the absence of any commercial or financial relationships that could be construed as a potential conflict of interest.
