# Supplementary figures and images for: Foot-and-Mouth Disease: Optimization, Reproducibility, and Scalability of High-Yield Production of Virus-Like Particles for a Next-Generation Vaccine
Source: Front Vet Sci. 2020 Sep 23;7:601. doi: 10.3389/fvets.2020.00601 (PMC7538550; doi:10.3389/fvets.2020.00601)

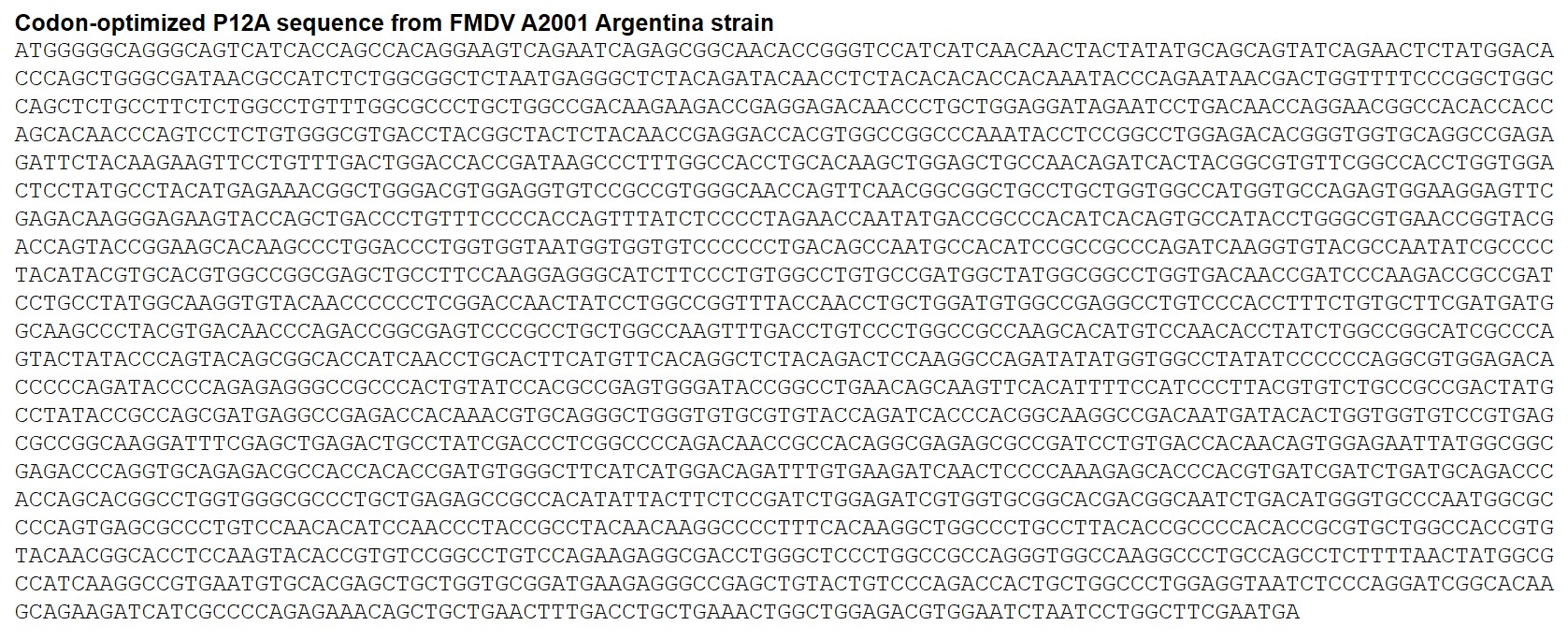

Supplement: Supplementary file 1 [file Image_1.JPEG]
